# Dynamic changes in macrophage populations and resulting alterations in Prostaglandin E_2_ sensitivity in mice with diet-induced MASH

**DOI:** 10.1186/s12964-025-02222-y

**Published:** 2025-05-16

**Authors:** Madita Vahrenbrink, C. D. Coleman, S. Kuipers, I. Lurje, L. Hammerich, D. Kunkel, J. Keye, S. Dittrich, B. M. Schjeide, R. Hiß, J. Müller, G. P. Püschel, J. Henkel

**Affiliations:** 1 Department of Nutritional Biochemistry, Institute of Nutritional Science, University of Potsdam, Nuthetal, Germany; 2 Max Rubner Center (MRC) for Cardiovascular Metabolic Renal Research, Institute of Pharmacology, Charité-Universitätsmedizin Berlin, Hessische Straße 3-4, 10115 Berlin, Germany; 3 Department of Hepatology and Gastroenterology, Charité-Universitätsmedizin Berlin, Berlin, Germany; 4 Flow & Mass Cytometry Core Facility, Berlin Institute of Health at Charité- Universitätsmedizin Berlin, Berlin, Germany; 5 Nutritional Biochemistry, Faculty of Life Sciences: Food, Nutrition and Health, University of Bayreuth, Kulmbach, Germany; 6 Physics and Computer Sciences, Applied Computer Sciences VIII, Faculty of Mathematics, University of Bayreuth, Bayreuth, Germany

**Keywords:** Cyclooxygenase, Prostaglandin E_2_, Kupffer cells, Infiltrating macrophages, Inflammation, MASH, TNF-α

## Abstract

**Background:**

The transition from metabolic dysfunction-associated steatotic liver disease (MASLD) to steatohepatitis (MASH) is characterized by a chronic low-grade inflammation, involving activation of resident macrophages (Kupffer cells; KC) and recruitment of infiltrating macrophages. Macrophages produce cytokines and, after induction of Cyclooxygenase 2 (COX-2), the key enzyme of prostanoid synthesis, prostaglandin E_2_ (PGE_2_). PGE_2_ modulates cytokine production in an autocrine and paracrine manner, therefore playing a pivotal role in regulating inflammatory processes. Changes in the hepatic macrophage pool during MASLD progression to MASH could influence PGE_2_- and cytokine-mediated signaling processes. The aim of this study was to characterize these changes in mice with diet-induced MASH and further elucidate the role of COX-2-dependently formed PGE_2_ on the inflammatory response in different macrophage populations of mice with a macrophage-specific COX-2-deletion.

**Methods:**

Male, 6-7-week-old wildtype mice were fed either a Standard or high-fat, high-cholesterol MASH-inducing diet for 4, 12 and 20 weeks. Liver macrophages were isolated and analyzed by flow cytometry. For in vitro experiments primary KC, peritoneal macrophages (PM) and Bone-marrow-derived macrophages (BMDM) were isolated from macrophage-specific COX-2-deficient and wildtype mice and treated with lipopolysaccharide (LPS) and/or PGE_2_.

**Results:**

During MASH-development, the proportion of KC (Clec4F^+^Tim4^+^) decreased, while the proportion of monocyte-derived macrophages (Clec4F^−^Tim4^−^) and monocyte-derived cells exhibiting a phenotype similar to KC (Clec4F^+^Tim4^−^) significantly increased over time. In vitro experiments showed that exogenous PGE_2_ completely abrogated the LPS-induced mRNA expression and secretion of tumor necrosis factor-alpha (TNF-α) in primary KC, PM and BMDM from wildtype mice. PM and BMDM, as in vitro models for infiltrating macrophages, were more sensitive to PGE_2_ compared to KC. Deletion of COX-2 in all macrophage populations led to an impaired PGE_2_-dependent feedback inhibition of TNF-α production. LPSinduced TNF-α mRNA expression was higher compared to the respective wildtype macrophage population.

**Conclusion:**

The current study, using a murine MASH model, indicates that PGE_2_ may have a protective, anti-inflammatory effect, especially by inhibiting the expression of pro-inflammatory cytokines such as TNFα in infiltrating monocyte-derived macrophages. An inhibition of endogenous PGE_2_ synthesis in macrophages by pharmacological inhibition of COX-2 could potentially increase inflammation and promote the progression of MASH.

**Supplementary Information:**

The online version contains supplementary material available at 10.1186/s12964-025-02222-y.

## Graphical abstract

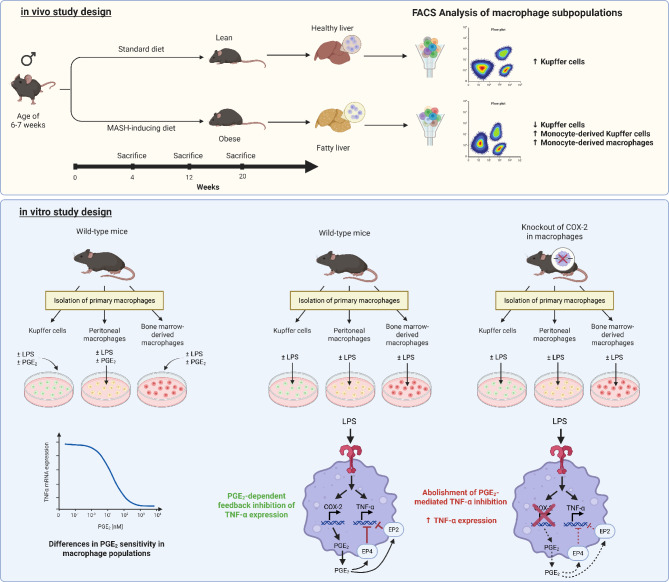

## Background

Metabolic dysfunction-associated steatotic liver disease (MASLD) is one of the most common liver diseases worldwide, associated with cardio-metabolic comorbidities such as obesity, hyperglycemia, hypertension and dyslipidemia [1, 2]. Among multiple other factors, MASLD might occur due to an increased dietary intake of fatty acids and cholesterol [3, 4]. The disease spectrum ranges from simple steatosis to steatohepatitis (MASH) and cirrhosis, with an increasing risk of developing hepatocellular carcinoma. The transition from benign steatosis to progressive MASH is characterized by hepatocyte damage, inflammatory processes and initial fibrosis [5, 6]. By contributing to the inflammatory response and by modulating it, resident and infiltrating macrophages are thought to play an essential role in driving MASLD progression [7, 8].

Kupffer cells (KC), the liver resident macrophages, and infiltrating macrophages are activated by danger-associated molecular patterns (DAMP) that are released by stressed or dying hepatocytes, as well as high concentrations lipopolysaccharide (LPS) in the portal circulation resulting from a pathologically increased permeability of the intestine [8, 9]. Following activation, macrophages produce a number of pro-inflammatory mediators, including the cytokine tumor necrosis factor-alpha (TNF-α), that can induce insulin resistance in hepatocytes and trigger hepatocyte apoptosis [10, 11]. Furthermore, the expression of cyclooxygenase 2 (COX-2) and microsomal prostaglandin E_2_ synthase-1 (mPGES-1) is up-regulated in activated macrophages, leading to the production of the small lipid mediator prostaglandin E_2_ (PGE_2_). PGE_2_ acts in an autocrine and paracrine manner and, among others, enhances the secretion of pro-inflammatory cytokines and chemokines such as oncostatin M (OSM) and interleukin-8 (IL-8) as demonstrated by our own data [12–14]. In parallel PGE_2_ inhibits the DAMP- and LPS-induced TNF-α expression in macrophages [15–17]. Thus, PGE_2_ could have potential pro- and anti-inflammatory functions in the context of MASH development. We have shown in a previous study that an impaired PGE_2_ synthesis in mPGES1-deficient mice with diet-induced MASH resulted in an enhanced TNF-α-mediated liver inflammation [18]. In addition, transgenic overexpression of COX-2 in livers was associated with reduced inflammation and partially protected mice from diet-induced MASH development [19].

Recent evidence suggests that the liver resident KC, which appear to orchestrate the inflammation in early phases of MASH, are replaced by infiltrating monocyte-derived macrophages during disease progression [7, 20]. These changes in the hepatic macrophage pool could possibly influence PGE_2_-dependent signaling processes in different phases of disease development. Therefore, the aim of this study was to characterize the macrophage pool in mice fed with a cholesterol-containing high-fat diet composed of a high content of ω6-polyunsaturated fatty acids, which evidently induces MASH accompanied by obesity and insulin resistance [21, 22], and to elucidate the role of PGE_2_-dependent regulation of cytokine expression in different macrophage populations. Taken together, our data suggests that differences in PGE_2_ sensitivity in macrophages may influence inflammatory processes in MASH progression.

## Materials and methods

All chemicals were of analytical or higher grade and obtained from local providers unless otherwise stated.

### Animals and experimental design

C57BL/6JRj mice expressing the Cre recombinase under control of the Lysozyme M gene (*Lyz*) [23] and COX-2-floxed mice [24] were bred to generate a macrophage-specific COX-2-deletion (COX-2^ΔMφ^). COX-2-floxed littermates without LysM Cre expression (COX-2^flox/flox^) or unfloxed mice either with or without a heterozygous LysM Cre (COX-2^+/+^ LysM Cre^+/−^, COX-2^+/+^) expression were used as wildtype controls. All mice were kept at 20 ± 2 °C with a 12 h light-dark cycle and with free access to food and water. For the feeding study, male wildtype mice at the age of 6–7 weeks were randomly assigned to standard diet (STD; V153 R/M-H; Ssniff, Soest, Germany) or a MASH-inducing high-fat, high-cholesterol diet containing soybean oil and 0.75% cholesterol (MASH-D [22]; Altromin, Lage, Germany) for 4, 12 or 20 weeks. Body weight was measured weekly. Mice were killed by cervical dislocation after isoflurane anesthesia. Importantly, no animal deaths were observed as a result of the prolonged MASH-D feeding in this or previous studies [18, 21, 22]. Animal experiments were performed according to the ARRIVE guidelines [25]. Treatment of the animals followed the German animal protection laws and was performed with permission of the state animal welfare committee (LUGV Brandenburg, 2347-43-2019).

### Hepatic histology and tissue analysis

Liver triglycerides and cholesterol were determined by colorimetric assay kits (HUMAN, Wiesbaden, Germany). Formalin-fixed and paraffin-embedded liver Sects. (2–3 μm) were stained with Hematoxylin & Eosin (Merck, Taufkirchen, Germany). Histological steatosis was quantified using CellProfiler (v.5.0.0 beta 1 (1)) in images of 10 randomly chosen fields of each liver section. For detection of lipid droplets, the Cellpose (v.2.2 (2)) segmentation algorithm was used and a custom segmentation model was trained. The measurements were analyzed in an iPython notebook available at the open research repository Zenodo using Panda (v.1.5.0) and statsmodels (v.0.13.2).

### Isolation and cultivation of murine Kupffer cells, peritoneal macrophages and bone marrow-derived macrophages

Cells were obtained from STD-fed male wildtype (KC: *n* = 33; PM: *n* = 27; BMDM: *n* = 30) or macrophage-specific COX-2-deficient mice (COX-2^ΔMφ^; KC: *n* = 10; PM: *n* = 8; BMDM: *n* = 9). Kupffer cells were isolated as previously described [21, 26]. Density gradient-purified Kupffer cells were cultured for 48 h in low-endotoxin RPMI medium (PAN- Biotech GmbH, Aidenbach, Germany) containing 1% penicillin (1000 U/mL) and streptomycin (100 µg/mL) (P/S) (PAN-Biotech) and 30% heat-inactivated fetal calf serum (PAN-Biotech). Peritoneal macrophages were isolated by peritoneal lavage with 3% fetal calf serum in phosphate-buffered saline [27] and cultured for 24 h in low-endotoxin RPMI medium containing 1% P/S and 10% heat-inactivated fetal calf serum, as well as 100 ng/mL phorbol-12-myristate-13-acetate (Sigma-Aldrich, Taufkirchen, Germany) for the first 2 h. Bone marrow cells were isolated by flushing femurs and tibiae with RPMI medium [28]. The cells were cultured at a density of 1,6 × 10^6^ cells/well in low-endotoxin RPMI supplemented with 1% P/S, 1% L-glutamine (PAN-Biotech), 0.25 µg/mL Amphotericin B (PAN-Biotech), 20% fetal calf serum and 10 ng/mL recombinant murine macrophage colony stimulating factor (M-CSF) (PeproTech GmbH, Hamburg, Germany) for 6 days. Macrophages were stimulated with 1 or 10 ng/mL lipopolysaccharide (LPS) from *Escherichia coli* (Serotype O55:B5; Sigma-Aldrich, Taufkirchen, Germany) and/or 1 µM Prostaglandin E_2_ (PGE_2_; Enzo Life Science GmbH, Lörrach, Germany) for 24 h.

### Flow cytometry analysis of non-parenchymal liver cells

Mouse livers were perfused with PBS, isolated, minced and digested with Collagenase IV (Worthington, Lakewood, NJ, USA) and DNAse I (Roche, Basel, Switzerland) at 37 °C. Extracts were filtered through a 70 μm mesh sieve and non-parenchymal liver cells were purified by density-gradient centrifugation. An appropriate amount of cells were resuspended in staining buffer (HBSS + 2 mM EDTA) and pre-incubated with Zombie NIR fixable viability dye (Biolegend, Inc., San Diego, CA, USA) followed by an incubation with specific fluorochrome-conjugated antibodies (see Supplementary table T1) at room temperature. All antibodies were diluted 1:400 in blocking buffer (PBS + 2% BSA + 2% mouse/rat/human/rabbit serum). Cells were then fixed for 10 min in 2% paraformaldehyde at room temperature and resuspended in staining buffer after a final washing step. To determine total cell numbers Precision Count beads (Biolegend, Inc., San Diego, CA, USA) were added as internal references prior to the measurement. Cell suspensions were analyzed using a Cytek^®^ Aurora Cytometer (Cytek Bioscience, Fremont, CA, USA) equipped with 3 lasers (405, 455 638 nm) and FCS Express Version 7.16.0035 (DeNovo Software, Pasadena, CA, USA). For gating strategy, see Supplementary figure S1.

### Real-time RT-PCR analysis

Cultured cells were washed with ice-cold PBS and frozen in liquid nitrogen. RNA was isolated using the ReliaPrep RNA Tissue Miniprep System (Promega GmbH, Walldorf, Germany). Reverse transcription and qPCR were performed as previously described [13]. Oligonucleotide sequences are listed in Supplementary table T2. Results are expressed as relative gene expression normalized to expression levels of the reference gene (*Hprt*) according to the formula: fold induction = 2 ^(control−treated) gene of interest^ / 2 ^(control−treated) reference gene^. The absolute quantification of EP receptors in macrophages was carried out using standard curves created with corresponding plasmids of each EP receptor subtype as well as the reference gene *Hprt* [12]. A quotient was then formed from the copy number of the EP receptors and the reference gene and multiplied by a factor of 100 for better visualization.

### Western blot analysis

Western blot was performed as described previously [29] with anti-COX-2 (#12282, Cell signaling, Heidelberg, Germany) and anti-β-Actin-HRP antibodies (#A3854, Sigma-Aldrich, Taufkirchen, Germany). Visualization of immune complexes was performed by using chemiluminescence reagent in ChemiDoc™ Imaging System with ImageLab software (Bio-Rad, Munich, Germany).

### Determination of PGE_2_ and TNFα

Cell culture supernatants were analyzed with enzyme-linked immunoassay kits for determination of PGE_2_ (Cayman Chemical, Ann Arbor, Michigan, USA) and TNFα (Life Technologies, Darmstadt, Germany) according to the manufacturer’s instructions.

### Statistical analysis

The statistical significance of differences was determined by Student’s t-test for unpaired samples and either One-way- or Two-way-ANOVA with Tukey’s *post hoc* test for multiple comparisons, as detailed in the legends to the figures, using GraphPad Prism version 8 for Windows (GraphPad Software, La Jolla California USA). Differences with a *p* ≤ 0.05 were considered statistically significant.

## Results

### Hepatic macrophage pool changed dynamically during MASH development

**Fig. 1:**
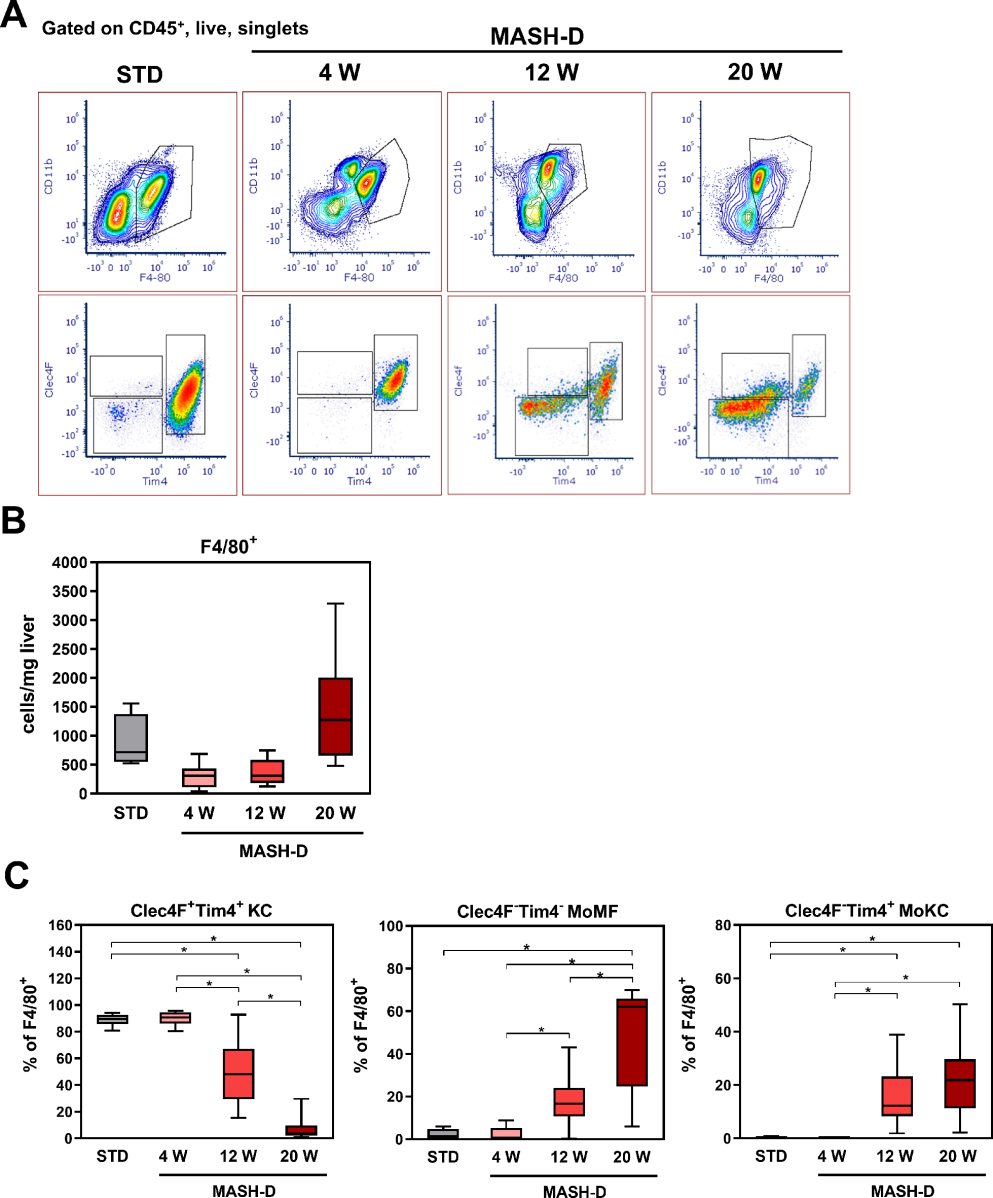
Dynamic changes in hepatic macrophage populations of mice with diet-induced MASH. (**A**) Representative density plots of liver macrophage populations at indicated feeding periods. After gating on CD45^+^, live and singlet cells, macrophages were identified as F4/80^+^ cells (top panel) and further analysed for expression of Tim4 and Clec4F (bottom panel). (**B**) Quantification of F4/80^+^ cells per milligram liver tissue. (**C**) Proportion of Clec4F^+^Tim4^+^ Kupffer cells (KC), Clec4F^−^Tim4^−^ monocyte-derived macrophages (MoMF), and Clec4F^+^Tim4^−^ monocyte-derived Kupffer cells (MoKC) as fractions of F4/80^+^ cells. Values are median (line), upper- and lower quartile (box) and extremes (whiskers) of *n* = 4–8 (STD), *n* = 8–11 (4 W MASH-D), *n* = 8–13 (12 W MASH-D), *n* = 9–11 (20 W MASH-D) mice. Statistics: One-way-ANOVA with Tukey´s *post hoc* test for multiple comparison. *p < 0.05

In order to assess the composition of the hepatic macrophage pool in the course of MASH development, mice were fed either a standard diet (STD) or a soybean-oil-based high-fat high-cholesterol diet (MASH-inducing diet, MASH-D). As described before, feeding of this specific MASH-D for 4 weeks was sufficient to induce hepatic steatosis in mice, whereas 20 weeks of feeding resulted in MASH with steatosis, inflammation and initial fibrosis [21, 22]. Apart from 4 to 20 weeks of feeding, an additional feeding period of 12 weeks was chosen, to evaluate the macrophage composition in an intermediate MASLD stage. At the end of each feeding period the non-parenchymal cells of livers from STD and MASH-D fed mice were isolated by enzymatic digestion of the liver and then analyzed by flow cytometry. Dead cells, cell doublets as well as non-myeloid cell populations such as T cells, NK cells, B cells and granulocytes were excluded (Supplementary figure S1), and total liver macrophages were identified as CD11b^+^ F4/80^+^ (Fig. 1A, top panel). Resident Kupffer cells (KC) specifically express the cell surface receptors C-type lectin domain family 4 member F (Clec4F) and T cell immunoglobulin and mucin domain containing 4 receptor (Tim4) and thus, can be distinguished from infiltrating monocyte-derived macrophages (MoMF), which do not express either of these markers (Fig. 1A, bottom panel) [20]. In addition to Clec4F^+^Tim4^+^ KC and Clec4F^−^Tim4^−^ MoMF, we were able to verify the presence of a third Clec4F^+^Tim4^−^ macrophage population, that infiltrates the liver and differentiates to KC (MoKC) which was previously described as monocyte-derived macrophages [20, 30]. The number of F4/80^+^ macrophages remained relatively constant over time, although a slight increase was detected after 20 weeks of MASH-D feeding (Fig. 1B). Similarly, the number of F4/80^+^ macrophages in the livers of STD-fed control mice did not change significantly over time (Supplementary Figure S2), suggesting that age-related fluctuations did not affect the number of F4/80 + macrophages over the course of feeding. The proportion of Clec4F^+^Tim4^+^ KC was significantly reduced after 12 weeks of MASH-D, and even further reduced after 20 weeks of MASH-D (Fig. 1C). Clec4F^−^Tim4^−^ MoMF were already present in mice fed a STD. Beginning from week 12 of MASH-D feeding, the proportion of MoMF significantly increased and nearly 20% of F4/80^+^ macrophages were Clec4F^−^Tim4^−^. After 20 weeks of MASH-D, Clec4F^−^Tim4^−^ MoMF accounted for 60% of total liver macrophages. Clec4F^+^Tim4^−^ MoKC were detectable only after 12 weeks of MASH-D, with their proportion being further increased after 20 weeks of MASH-D. Overall, the macrophage pool in the liver changes dynamically when fed a MASH-inducing diet, with the proportion of resident macrophages decreasing and the number of infiltrating macrophages increasing over time.

In line with disease progression, the number of lipid droplets increased to an average of 109 ± 13 per image after 4 weeks of MASH-D feeding, with no further significant changes at later time points (data not shown). In contrast, the average size of the lipid droplets continued to increase, reaching higher levels after 20 weeks compared to 4 and 12 weeks of MASH-D feeding (Supplementary Figure S3). Hepatic triglyceride and cholesterol levels were significantly elevated after 4, 12, and 20 weeks of MASH-D feeding compared to STD controls (Supplementary Table T3). Notably, triglyceride content was significantly higher after 12 and 20 weeks of feeding compared to 4 weeks, whereas cholesterol levels remained stable across the MASH-D time points. These findings suggest that progressive hepatic lipid accumulation, particularly the increase in triglyceride content and lipid droplet size, may contribute to the dynamic shift in the hepatic macrophage pool observed during MASH development. The decline in resident Kupffer cells and the concurrent rise in infiltrating monocyte-derived macrophages could reflect a compensatory response to sustained lipotoxic stress and inflammation in the liver microenvironment. This supports the notion that altered lipid homeostasis plays a central role in shaping macrophage composition and function in MASH.

**Fig. 2:**
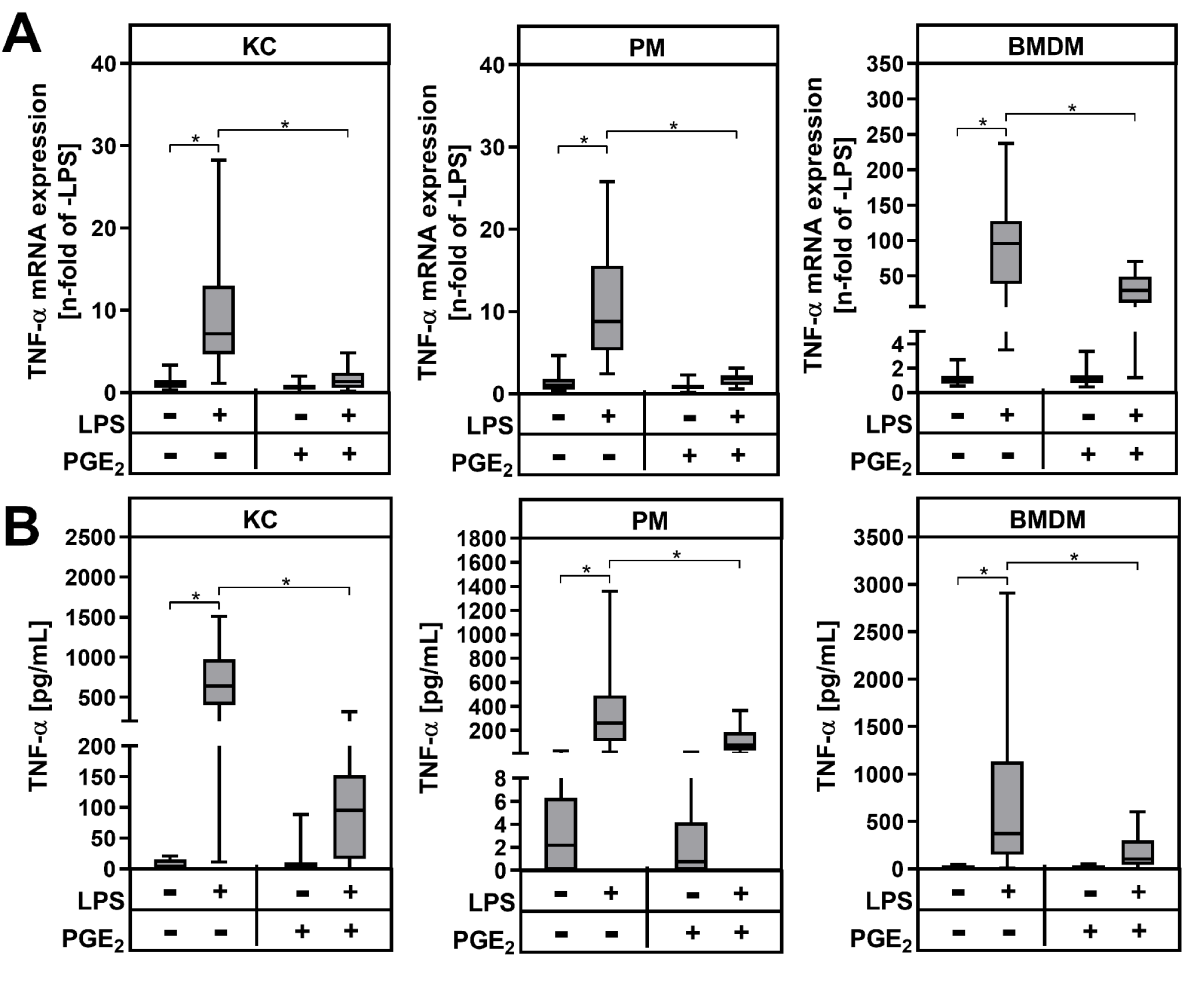
Modulation of TNF-α mRNA and protein expression by exogenous PGE_2_. Primary Kupffer cells (KC), peritoneal macrophages (PM) and bone marrow-derived macrophages (BMDM) were stimulated for 24 h with LPS and/or PGE_2_. (**A**) Relative mRNA expression of TNF-α was determined by RT-qPCR with *Hprt* as the reference gene. (**B**) TNF-α protein levels were quantified by ELISA in cell culture supernatants. Values are median (line), upper- and lower quartile (box) and extremes (whiskers) of (A) *n* = 33 (KC), *n* = 26 (PM) or *n* = 29 (BMDM) or (B) *n* = 27 (KC), *n* = 26 (PM) or *n* = 26 (BMDM) independent experiments. Statistics: Two-way-ANOVA with Tukey´s *post hoc* test for multiple comparison. *p < 0.05

### Cytokine expression is modulated differently by prostaglandin E_2_ according to macrophage populations

**Fig. 3:**
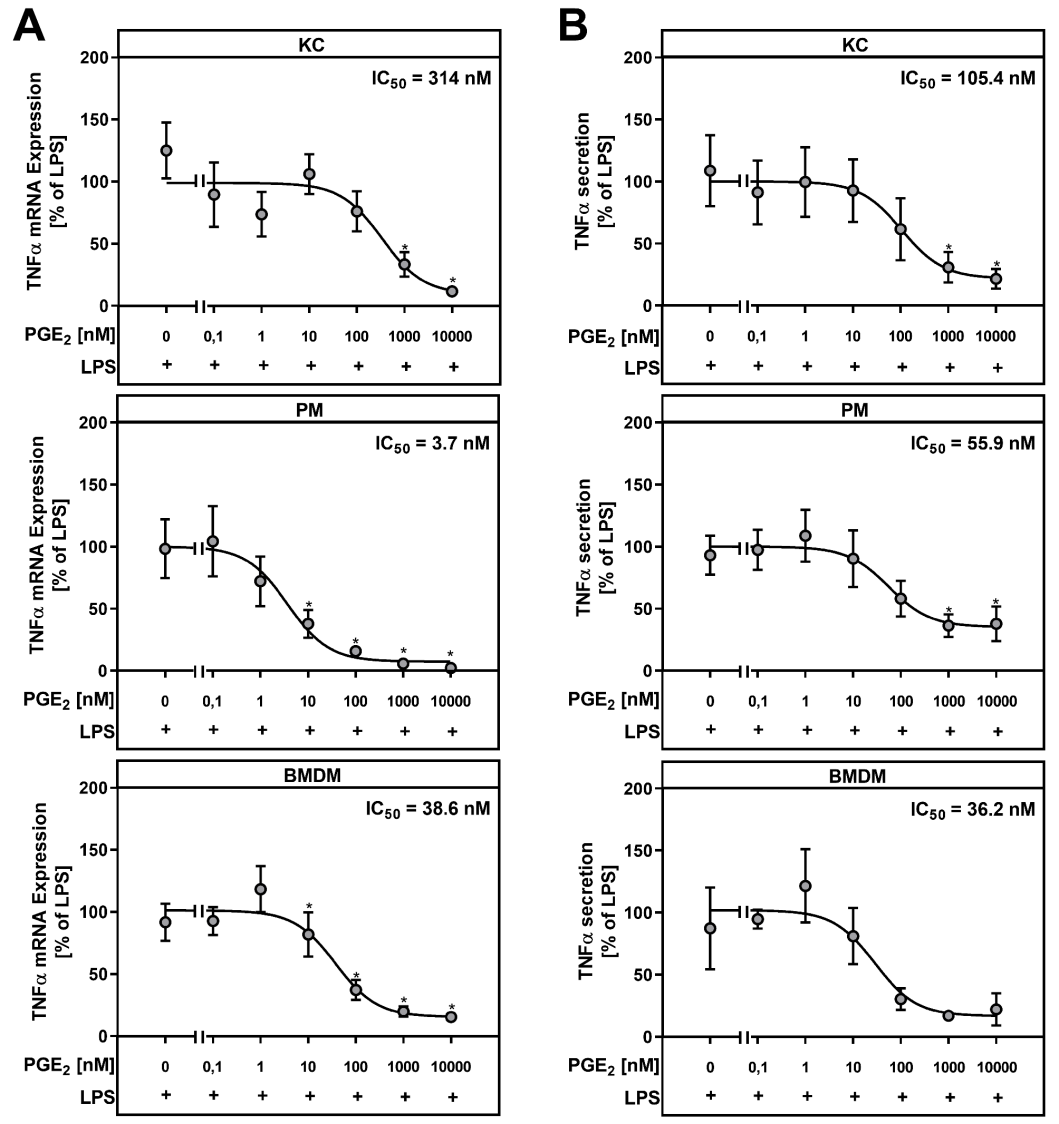
PGE_2_ inhibits TNF-α mRNA and protein expression in a dose-dependent manner. Primary Kupffer cells (KC), peritoneal macrophages (PM) and bone marrow-derived macrophages (BMDM) were stimulated for 24 h with LPS and indicated concentrations of PGE_2_. (**A**) Relative mRNA expression of TNF-α was determined by RT-qPCR with *Hprt* as the reference gene. (**B**) TNF-α protein levels were determined by ELISA in cell culture supernatants. Values are mean ± SEM of *n* = 5 independent experiments. Statistics: Student´s t-test for unpaired samples. * vs. w/o PGE_2_ with *p* < 0.05

To investigate how different macrophage populations react to PGE_2_, primary macrophages were isolated from wildtype (WT) mice and treated with lipopolysaccharide (LPS) and/or exogenous PGE_2_. The concentration of LPS required to activate the different macrophage populations was determined by dose-response curves (not shown). These indicated that 1 ng/mL LPS was sufficient for the activation of KC and PM, while 10 ng/mL was necessary for the activation of BMDM. Apart from liver resident KC, peritoneal macrophages (PM) and bone marrow-derived macrophages (BMDM) were used as models for infiltrating monocyte-derived macrophages. Treatment of KC, PM and BMDM with LPS significantly induced the expression of tumor necrosis factor-α (TNF-α) in all three macrophage populations (Fig. 2A). The combined incubation of primary macrophages with both LPS and PGE_2_ resulted in the inhibition of LPS-dependent TNF-α mRNA expression, while PGE_2_ alone had no effect on basal TNF-α mRNA expression. In line with the gene expression data, TNF-α protein levels in cell culture supernatants of KC, PM and BMDM, were significantly increased by LPS, whereas additional PGE_2_ treatment inhibited the LPS-dependent increase of TNF-α protein levels (Fig. 2B). The PGE_2_ concentration required to inhibit the LPS-induced TNF-α mRNA expression by 50% (half-maximal inhibitory concentration; IC_50_) was approximately 3.7 nM in PM and 38.6 nM in BMDM (Fig. 3A). In contrast to models for infiltrating macrophages, the IC_50_ for PGE_2_-mediated inhibition of LPS-induced TNF-α mRNA expression was 314 nM in the resident Kupffer cells. In parallel, the LPS-induced TNF-α secretion was half-maximally inhibited at much lower PGE_2_ concentrations in PM and BMDM compared to KC (Fig. 3B). Thus, BMDM were significantly more sensitive in terms of PGE_2_-dependent inhibition of TNF-α expression and secretion than KC, but not as sensitive as PM.

**Fig. 4:**
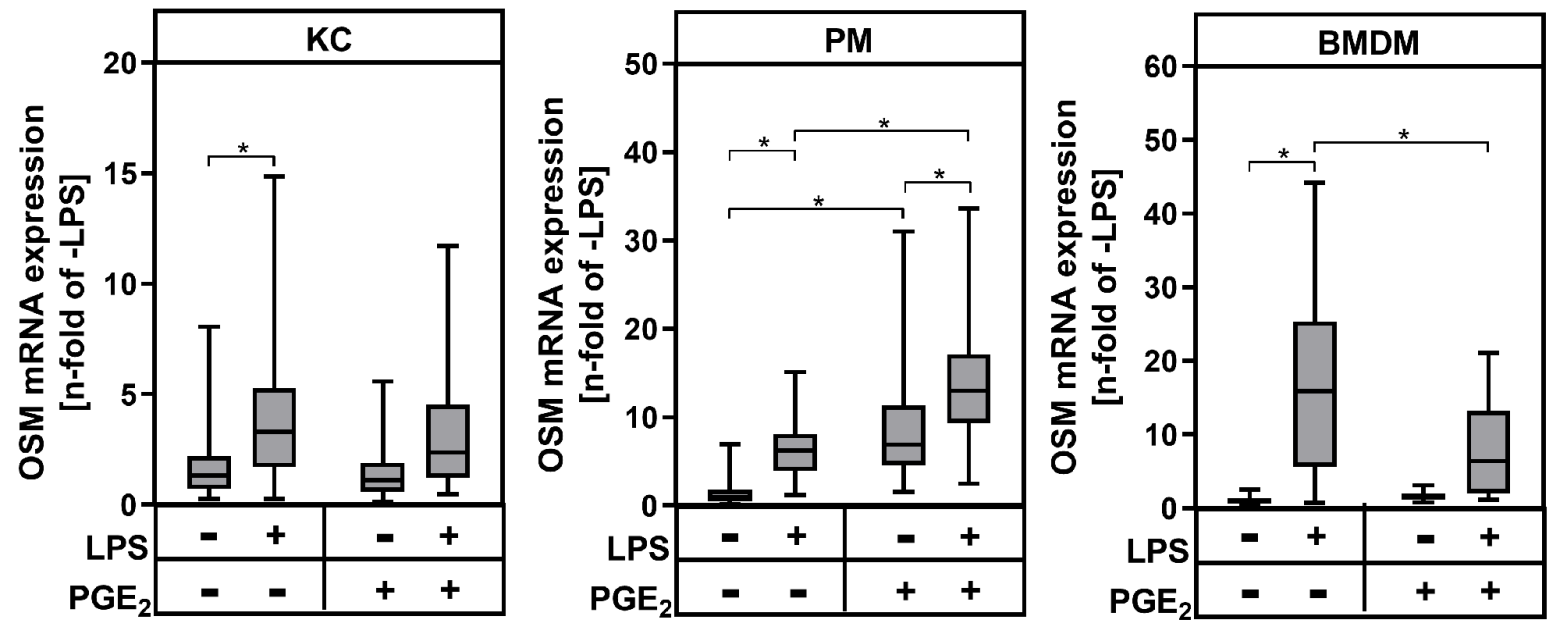
Modulation of OSM mRNA expression by exogenous PGE_2_. Primary Kupffer cells (KC), peritoneal macrophages (PM) and bone marrow-derived macrophages (BMDM) were stimulated for 24 h with LPS and/or PGE_2_. Relative mRNA expression of OSM was determined by RT-qPCR with *Hprt* as the reference gene. Values are median (line), upper- and lower quartile (box) and extremes (whiskers) of *n* = 28 (KC), *n* = 26 (PM) or *n* = 30 (BMDM) independent experiments. Statistics: Two-way-ANOVA with Tukey´s *post hoc* test for multiple comparison. *p < 0.05

Furthermore, we aimed to assess the PGE_2_-dependent regulation of other pro-inflammatory cytokines. We have previously shown that the pro-inflammatory cytokine oncostatin M (OSM) may contribute to development of hepatic insulin resistance and steatosis and that its production is enhanced by PGE_2_ in primary rat KC [13]. In mouse KC only LPS, but not PGE_2_, induced the OSM mRNA expression (Fig. 4). In PM, however, both LPS and PGE_2_ induced OSM mRNA expression and PGE_2_ synergistically enhanced the LPS-induced OSM mRNA expression. Surprisingly, PGE_2_ inhibited the LPS-induced OSM mRNA expression in BMDM, while not effecting the basal cytokine expression.

PGE_2_ mediates its effect via four membrane-bound prostaglandin E_2_ receptors (EP) [31, 32]. Therefore, we next quantified the expression of all four EP receptor subtypes in primary macrophages and we could observe that PM and BMDM primarily expressed both of the G_s_-coupled EP2 and EP4 receptors, followed by the G_q_-coupled EP1 receptor and, with the lowest expression, G_i_-coupled EP3 receptor (Table 1). In primary KC the EP2 receptor was also most highly expressed, but in contrast to PM and BMDM, the expression of the EP1 receptor was higher than that of the EP4 receptor. The EP1 receptor expression did not differ between the three macrophage populations. However, PM had a significantly higher EP2 receptor expression compared to KC and BMDM. Moreover, the EP4 receptor expression tended to be higher in BMDM compared to KC, with the highest expression in PM. EP3 receptor expression was lowest in all macrophage subtypes (Table 1). These observations suggest that differences in EP receptor subtype expression in KC, PM and BMDM might be responsible for their different reaction to exogenous PGE_2_.

**Table 1:**
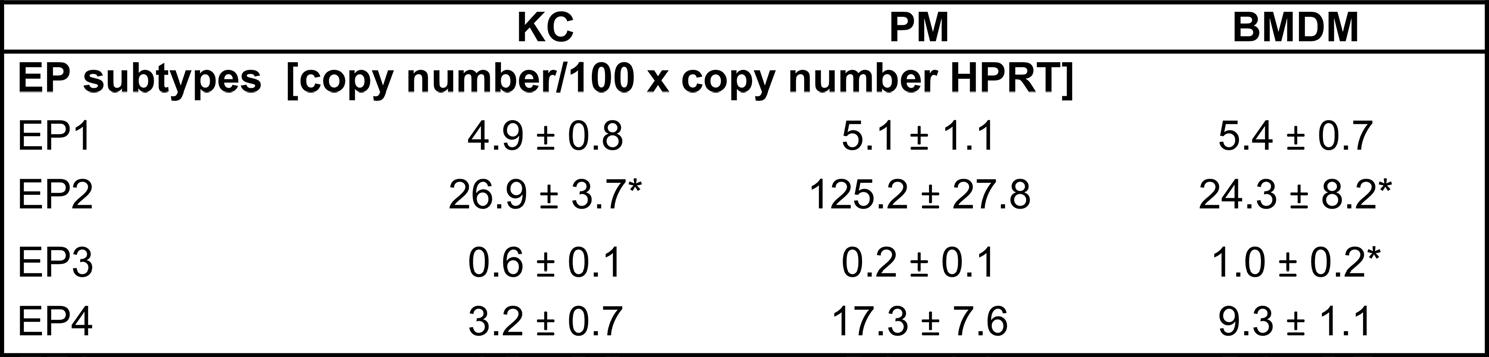
Expression of Prostaglandin E_2_ receptor (EP) subtypes in macrophage populations. Absolute copy numbers of EP receptor subtypes in Kupffer cells (KC), peritoneal macrophages (PM) and bone marrow-derived macrophages (BMDM) were quantified in relation to *Hprt* by RT-qPCR. Values are mean ± SEM of *n* = 5 (KC, BMDM) or *n* = 7 (PM) independent experiments. Statistics: One-way-ANOVA with Tukey´s *post hoc* test for multiple comparison. * vs. PM with *p* < 0.05.

### PGE_2_-mediated feedback loops were impaired in mice with macrophage-specific COX-2-deficiency

**Fig. 5:**
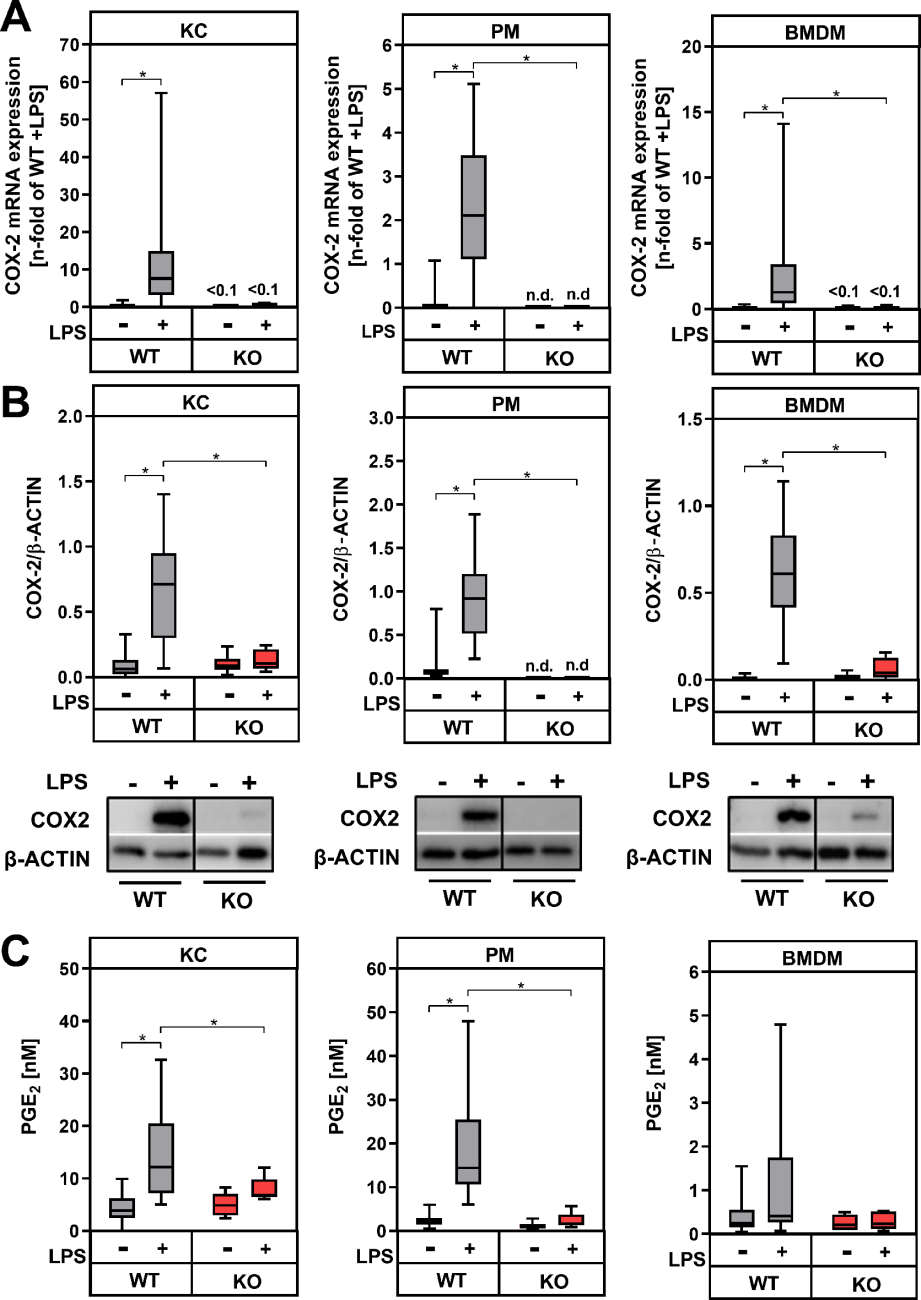
Modulation of LPS-mediated expression of cyclooxygenase 2 (COX-2) and secretion of prostaglandin E_2_ (PGE_2_) in macrophages from wildtype controls (WT) and COX-2-deficient mice (KO). Primary Kupffer cells (KC), peritoneal macrophages (PM) and bone marrow-derived macrophages (BMDM) were stimulated for 24 h with LPS. (**A**) Relative mRNA expression of COX-2 (gene name *Ptges2*) was determined by RT-qPCR with *Hprt* as the reference gene. (**B**) Protein lysates were analyzed by immunoblotting for COX-2 protein expression, with β-Actin serving as the loading control. All original blots are provided in Supplementary figure S5-7. (**C**) PGE_2_ level were determined by ELISA in cell culture supernatants. Values are median (line), upper- and lower quartile (box) and extremes (whiskers) of *n* = 26–32 (WT) or *n* = 5–10 (KO) independent experiments. Statistics: Two-way-ANOVA with Tukey´s *post hoc* test for multiple comparison. *p < 0.05. n.d.: not detectable

**Fig. 6:**
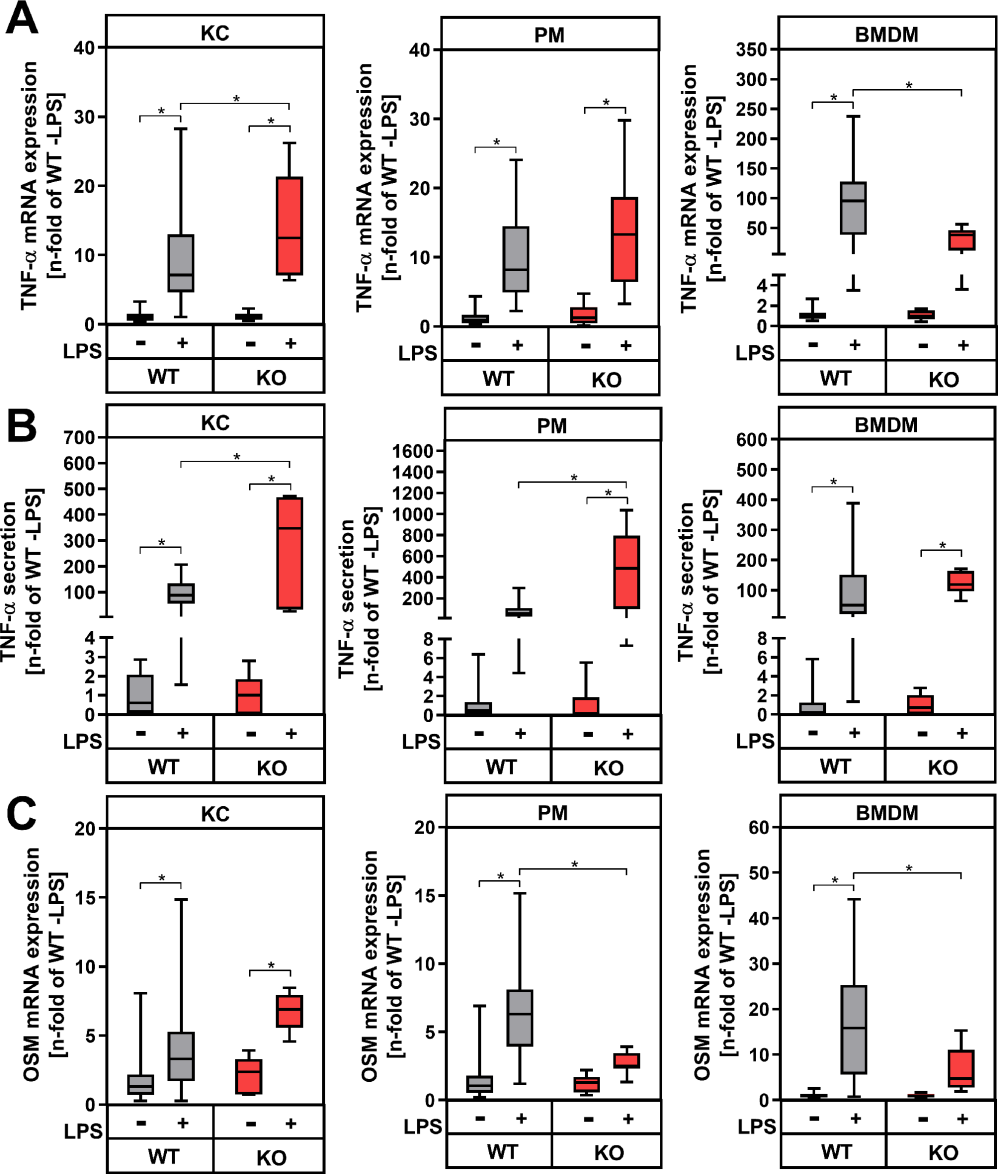
Modulation of cytokine mRNA and protein expression in macrophages from wildtype controls (WT) and COX-2-deficient mice (KO). Primary Kupffer cells (KC), peritoneal macrophages (PM) and bone marrow-derived macrophages (BMDM) were stimulated for 24 h with LPS. Relative mRNA expression of TNF-α (**A**) and OSM (**C**) were determined by RT-qPCR with *Hprt* as the reference gene. (**B**) TNF-α protein levels were determined by ELISA in cell culture supernatants. Values are median (line), upper- and lower quartile (box) and extremes (whiskers) of *n* = 26–32 (WT) or *n* = 5–10 (KO) independent experiments. Statistics: Two-way-ANOVA with Tukey´s *post hoc* test for multiple comparison. *p < 0.05

LPS significantly induced the mRNA and protein expression of the PGE_2_-synthesizing enzymes COX-2 (Fig. 5A, B) and mPGES1 (Supplementary figure S4) in macrophage populations from wildtype control mice. Accordingly, endogenous PGE_2_ synthesis was up-regulated in KC, PM and BMDM (Fig. 5C). While in KC medium, a basal PGE_2_ concentration of about 4.5 nM was measured, which was increased 4fold after LPS stimulation, PM basally released only about 2.6 nM PGE_2_ into the medium. After stimulation with LPS, the PGE_2_ concentration in cell culture supernatants of PM increased to 20 nM. By contrast, PGE_2_ secretion in BMDM was much lower and the LPS-dependent increase did not reach statistical significance, despite 10-fold higher LPS concentration used for stimulation. Macrophages isolated from tissue-specific COX-2-deficient mice with LysM-dependent expression of Cre recombinase (COX-2^ΔMφ^, KO) did not show any significant COX-2 mRNA or protein expression (Fig. 5A, B). While COX-1 expression was slightly upregulated in KC from COX-2 deficient mice compared to controls, no genotype-dependent differences were detected in PM and BMDM (Supplementary fig. S4A). In addition, LPS-dependent induced mPGES-1 expression was not, or only slightly, modified in COX-2 deficient macrophages compared to wildtype controls (Supplementary fig. S4B). PGE_2_ secretion from macrophages with COX-2 deficiency was significantly reduced to basal levels (Fig. 5C). The reduced endogenous PGE_2_ synthesis was accompanied by a significantly higher LPS-dependent induction of TNF-α mRNA expression in KC from COX-2-deficient mice compared to wildtype controls (Fig. 6A). In PM from COX-2-deficient mice, the LPS-induced TNF-α mRNA expression also tended to be higher compared to wildtype controls, while COX-2-deficient BMDM unexpectedly showed a significantly lower LPS-induced TNF-α mRNA expression than wildtype BMDM. Similar to gene expression data, a significantly higher LPS-induced TNF-α secretion could be observed in both COX-2-deficient KC and PM compared to wildtype macrophages (Fig. 6B). On the other hand, the LPS-induced TNF-α secretion did not differ between genotypes in BMDM. The basal OSM mRNA expression was comparable between macrophages from COX-2-deficient and wildtype mice (Fig. 6C). LPS-induced OSM mRNA expression was not affected by COX-2-deficiency in KC, however it was significantly inhibited in COX-2-deficient PM and BMDM compared to wildtype controls. Overall, COX-2-deficiency resulted in an impaired PGE_2_-mediated feedback inhibition of TNF-α gene expression and secretion in KC and PM, but not in BMDM. Furthermore, endogenously synthesized PGE_2_ is sufficient to mediate a feed forward amplification of OSM expression in PM. These data suggest that endogenously synthesized PGE_2_ might either dampen inflammation by inhibiting TNF-α production or amplify it by increasing OSM expression in macrophages.

## Discussion

The current study showed dynamic remodeling of the hepatic macrophage pool during MASLD progression, with an increased proportion of infiltrating macrophages and a decreased proportion of liver resident Kupffer cells (KC). Additional in vitro experiments highlight differential responses of macrophage populations to prostaglandin E_2_ (PGE_2_), implicating a possible role of PGE_2_ as an important modulator of inflammation during MASH development. However, certain limitations must be considered when interpreting these findings. Although our in vitro experiments using primary KC, peritoneal macrophages (PM) and bone marrow-derived macrophages (BMDM) were essential to dissect cell-specific responses to PGE_2_ in different macrophage populations under controlled conditions, the in vitro systems cannot fully recapitulate the complexity of the hepatic microenvironment. Factors such as local cytokine gradients, cell–cell interactions, and tissue architecture are absent in culture, which may influence the responsiveness of macrophages in vivo [33]. Moreover, while PM are commonly used as a surrogate for monocyte-derived infiltrating macrophages, their phenotypic features significantly differ from these cells [34, 35]. The same applies for BMDM, which are extensively used in in vitro studies, but show a distinct functional phenotype compared to macrophages infiltrating the MASH liver [36]. To overcome these limitations and strengthen the data obtained from the in vitro studies, we originally intended to use the sorted KC, MoMF and MoKC from healthy and MASH livers for characterization of PGE_2_ sensitivity and PGE_2_-dependent modulation of macrophage cytokine expression. However, due to the low yield of hepatic macrophage subpopulations, particularly KC and MoKC, following FACS-based isolation, it was not feasible to obtain sufficient numbers of healthy cells for downstream functional assays. For future studies, pooling cells obtained from several mice might be an option to investigate PGE_2_-mediated effects in sorted macrophage populations. An alternative approach, linking in vitro and in vivo data, would have been to isolate KC from mice fed either a STD or MASH-inducing diet, which was beyond the scope of the current animal study for which the use of a limited number of mice was authorized. Taking these limitations into account, our findings still reveal important insights regarding cellular mechanisms that are potentially relevant for MASLD progression and set the stage for more refined in vivo studies in the future.

The progression of MASLD from simple steatosis to MASH is accompanied by chronic inflammation, which is orchestrated by hepatic macrophages [8, 9, 37]. Danger-associated molecular patterns (DAMP) released by stressed or dying hepatocytes and elevated lipopolysaccharides (LPS) passing through the portal circulation activate tissue-resident Kupffer cells (KC), which mediate the recruitment of immune cells into the inflamed tissue [7–9, 38]. Among these immune cells are predominately monocytes from the bone marrow, which acutely differentiate into macrophages (bone marrow-derived macrophages; BMDM) [39]. However, there is evidence that more mature macrophage populations, such as macrophages from the peritoneum (peritoneal macrophages; PM), may also infiltrate the liver under these conditions [35]. Although a massive infiltration of macrophages into the liver during MASH development is described [7] the number of F4/80^+^ macrophages was only slightly elevated after 20 weeks of MASH-D feeding (Fig. 1B). This might be explained by dynamic changes within the hepatic macrophage pool. Apart from a small proportion of Clec4F^−^Tim4^−^ macrophages that were already detectable in STD-fed mice and most likely represent liver capsular macrophages rather than infiltrating macrophages (MoMF) [40], only resident Clec4F^+^Tim4^+^ KC were detectable in healthy livers (Fig. 1C). After 12 weeks of MASH-D feeding, the proportion of Clec4F^+^Tim4^+^ KC declines, while at the same time the proportion of Clec4F^−^Tim4^−^ MoMF macrophages increases (Fig. 1C). In addition, Clec4F^+^Tim4^−^ cells, previously described as monocyte-derived infiltrating macrophages with a KC similar phenotype (MoKC) [20], could be detected after 12 weeks of MASH-D (Fig. 1C). In line with this, a loss of Tim4^+^ KC and a simultaneous compensatory increase in Tim4^−^ MoMF has also been reported after 16 weeks of high-fat diet (HFD) [41]. However, others observed an increase of Clec4F^−^Tim4^−^ MoMF and Clec4F^+^Tim4^−^ MoKC already after 24 weeks of HFD feeding and a loss of Clec4F^+^Tim4^+^ KC only after 36 weeks of HFD resulting a transient increase of F4/80^+^ macrophages [42]. All in all, the proportion of Clec4F^+^Tim4^+^ KC decreased, while the proportion of infiltrating Clec4F^−^Tim4^−^ MoMF and Clec4F^+^Tim4^−^ MoKC increased during MASH development. Previous studies repeatedly described a ‘macrophage disappearance reaction’ under non-homeostatic conditions, meaning a diminished size of the KC pool accompanied by an increase of infiltrating monocyte-derived macrophages [20, 43]. The reason for KC death is poorly understood, although a recent publication indicated that Hypoxia inducible factor 2 alpha expression might promote KC death by inducing lysosomal stress [44]. Following KC loss, signals from hepatic stellate cells and endothelial cells stimulate the differentiation of monocyte-derived macrophages to MoKC, replenishing the resident macrophage niche [30, 42]. Additionally, changes in gene expression may lead to a loss of KC identity markers and ultimately cell death [45]. Macrophages adapt a distinct phenotype according to environmental signals [46]. In this context, the lipid-rich microenvironment induced by sustained MASH-D feeding (Supplementary figure S3) may contribute to the depletion of resident KCs and the recruitment and differentiation of MoMF into MoKC, as reflected by a shift from Clec4F⁺Tim4⁺ KCs to Clec4F⁻Tim4⁻ and Clec4F⁺Tim4⁻ populations. This is supported by our observation of increased MoMF and MoKC abundance after 12 and 20 weeks of MASH-D feeding (Fig. 1C). Furthermore, the strong rise in hepatic triglyceride content (Supplementary table T8) likely reflects lipotoxic stress that could alter KC gene expression, downregulate identity markers such as Tim4, and impair macrophage functions including phagocytosis, while also promoting a more pro-inflammatory macrophage phenotype [45, 46].

Activated resident Kupffer cells and newly recruited infiltrating macrophages produce pro-inflammatory cytokines and are the main sources of hepatic prostaglandin E_2_ (PGE_2_) [18]. In addition to liver resident KC and bone marrow-derived macrophages (BMDM), we included peritoneal macrophages (PM), a macrophage population well studied and often used as model for infiltrating monocyte-derived macrophages, in our in vitro experiments to elucidate the impact of PGE_2_ on cytokine expression in different macrophage populations. Upon stimulation with LPS, wildtype KC, PM and BMDM produced pro-inflammatory cytokines, such as TNF-α (Fig. 2) and OSM (Fig. 4). Furthermore, the PGE_2_-synthesizing enzymes COX-2 (Fig. 5A, B) and mPGES-1 (Supplementary fig. S4B) were significantly up-regulated in all three macrophage populations from wildtype mice following LPS treatment, resulting in an increased PGE_2_ release (Fig. 5C). PGE_2_ is known to either enhance or inhibit the inflammatory response by modulating the release of cytokines and chemokines. Treatment of KC, PM and BMDM with exogenous PGE_2_ led to a significant decrease in both LPS-dependent TNF-α gene and protein expression (Fig. 2). This anti-inflammatory effect of PGE_2_ has already been demonstrated repeatedly and across species in primary macrophages from rat [47, 48] and mouse [26, 49–52], as well as in human macrophages [17, 53]. In contrast to TNF-α, the expression of OSM can be induced by PGE_2_, as previously shown in rat KC [13]. We observed a similar effect of PGE_2_ in mouse PM, where exogenous PGE_2_ significantly induced the basal OSM mRNA expression and additionally increased the LPS-dependent OSM mRNA expression. On the other hand, OSM mRNA expression in KC was not affected by PGE_2_ and LPS-dependent OSM mRNA expression in BMDM was suppressed after PGE_2_ treatment (Fig. 4). An increased OSM expression and release from macrophages might lead to a disrupted insulin signaling in hepatocytes and promote hepatic lipid accumulation [13]. This effect might be further enhanced by an OSM-mediated induction of PGE_2_ synthesizing enzymes [54]. Thus, induction of OSM expression may represent a more pro-inflammatory function of PGE_2_.

PGE_2_ mediates its effect in an autocrine or paracrine manner via four prostaglandin E_2_ receptors (EP1-4) that are expressed in a cell specific manner [31]. Thus, the differences observed in the PGE_2_-dependent modulation of cytokine and chemokine expression between mouse KC, PM and BMDM may be due to differential expression of EP receptor subtypes. Other studies already reported that KC express all four EP receptor subtypes [26, 32], while only EP2 and EP4 were detectable in PM [55–57] and macrophage cell lines [58, 59]. In this study, we were able to detect all four receptor subtypes in primary mouse KC, PM and BMDM (Table 1). Consistent with the studies mentioned above, mouse PM and BMDM primarily expressed EP2 receptor and EP4 receptor, followed by EP1 receptor and the least expressed EP3 receptor. KC also expressed the EP2 receptor most strongly, but in contrast to PM and BMDM, the expression of the EP1 receptor was higher in these cells than that of the EP4 receptor. Direct comparison of EP expression between all three macrophage populations showed that PM and BMDM had higher EP2 and EP4 expression than KC, with the highest detected expression of both receptors in peritoneal macrophages. It has previously been shown that the LPS-induced TNF-α expression in macrophages was inhibited by treatment using EP2 and EP4 agonists to the same extent as by exogenous PGE_2_, whereas treatment using EP1 and EP3 agonists had no effect [26, 48, 52, 55, 57]. This difference in response, suggests that both G_s_-coupled receptor subtypes are predominantly responsible for this anti-inflammatory effect of PGE_2_. The binding affinity of PGE_2_ towards EP2 is described with a dissociation constant (K_D_) of 12–24 nM, whereas the binding of PGE_2_ to EP4 occurs with significantly higher affinity (K_D_=1–2 nM) [31, 60]. Accordingly, low PGE_2_ concentrations might be sufficient for inhibition of LPS-induced TNF-α expression via EP4, while inhibition of LPS-induced TNFα expression via EP2 is probably only relevant at much higher PGE_2_ concentrations [26]. In PM, a PGE_2_ concentration of 3.7 nM was sufficient for the half-maximal inhibition of the LPS-induced TNFα gene expression, whereas in BMDM and KC an approximately 10- and 100-fold higher PGE_2_ concentration was required for the same effect (Fig. 3A). For the inhibition of TNF-α secretion, KC required about twice the PGE_2_ concentration compared to PM and BMDM (Fig. 3B). The different sensitivities of the three macrophage populations to PGE_2_ could possibly be explained by the fact that EP4, in contrast to EP2, is desensitized and degraded faster as a result of agonist binding [48, 57, 61]. This could especially be true for KC, which are already exposed to higher basal PGE_2_ concentrations (approx. 4.5 nM) than PM and BMDM (Fig. 5C). Desensitization of EP4 receptor occurs rapidly after 10 min of agonist treatment [61]. Therefore, the observed PGE_2_-dependent modulation of cytokine expression in primary macrophages over a 24-h treatment period might be mainly mediated via EP2. In addition, the chosen time frame for our in vitro experiments might be too short for regeneration of EP4.

Cyclooxygenases (COX) are key enzymes in the generation of PGE_2_ and often targets for pharmacological inhibition to treat fever, pain and inflammation. Next to non-steroidal anti-inflammatory drugs (NSAID), which inhibit both COX isoforms, specific COX-2 inhibitors were discussed in treatment of several inflammatory diseases, but on the other hand were associated with a number of side effects, particularly affecting blood pressure and gastrointestinal integrity [62, 63]. Therefore, we used macrophages from mice with a macrophage-specific COX-2-deficiency and corresponding wildtype controls as a model to assess functionality of PGE_2_-mediated autocrine feedback regulation of cytokine production in primary macrophages. Consistent with other studies [64–66], the expression of the PGE_2_-synthesizing enzymes COX-2 and mPGES-1 was acutely upregulated in an LPS-dependent manner in KC and PM from wildtype mice (Fig. 5A, B, Supplementary Fig. 4B), followed by an increase of PGE_2_ concentrations in the cell culture supernatants of KC (17 nM) and PM (20 nM) (Fig. 5C). However, in wildtype BMDM, the LPS-induced expression of both enzymes did not lead to increased endogenous PGE_2_ synthesis (Fig. 5, Supplementary Fig. 4B). Nevertheless, the LPS-induced PGE_2_ synthesis was almost completely blunted in COX-2-deficient PM and BMDM and strongly reduced in COX-2-deficient KC compared to wildtype macrophages (Fig. 5C). Interestingly, COX-1 expression was slightly upregulated in COX-2-deficient KC compared to controls (Supplementary fig. S4A), while LPS-dependent induced mPGES-1 expression was not modified (Supplementary fig. S4B). Even though mPGES-1 mainly converts COX-2-dependently formed prostaglandin H_2_ (PGH_2_) to PGE_2_ [67–69], inhibition of endogenous PGE_2_ synthesis using a pharmacological COX-2 inhibitor was partially compensated by mPGES-1 utilizing COX-1-dependently formed PGH_2_ for PGE_2_ synthesis [65]. This could possibly explain why endogenous PGE_2_ synthesis was not completely abrogated in COX-2-deficient KC.

Since PGE_2_ inhibits LPS-induced TNF-α expression, we expected enhanced TNF-α expression in COX-2-deficient macrophages due to the impaired endogenous PGE_2_ synthesis. In accordance with this, the LPS-induced TNF-α mRNA and protein expression was significantly higher in COX-2-deficient KC and PM compared to wildtype macrophages (Fig. 6A, B). Similarly, pharmacological inhibition of PGE_2_ synthesis by the non-selective COX inhibitor indomethacin resulted in increased TNF-α secretion in primary macrophages [17, 26, 53]. Thus, the PGE_2_-dependent autocrine feedback inhibition of TNF-α expression is functional in KC and PM. In addition, PGE_2_ enhanced the LPS-induced OSM mRNA expression in an autocrine feedforward loop, as indicated by a significantly down-regulated LPS-induced OSM mRNA expression in COX-2-deficient PM (Fig. 6C). This points towards a potential pro-inflammatory effect of PGE_2_. In contrast to KC and PM, COX-2-deficient BMDM showed significantly lower LPS-induced TNF-α mRNA expression compared to wildtype cells (Fig. 6A), while TNF-α protein levels did not differ between genotypes (Fig. 6B). Again, other studies showed an increased TNFα secretion in BMDM treated with indomethacin [70, 71], suggesting that the autocrine feedback inhibition loop might also be active in BMDM. We have previously reported that impaired endogenous PGE_2_ synthesis caused by a global mPGES-1 KO resulted in elevated hepatic TNF-α levels and augmented liver inflammation in mice with diet-induced MASH [18]. This was most likely due to the disrupted PGE_2_-dependent feedback inhibition of TNF-α expression in macrophages, especially in infiltrating macrophages that react more sensitively to PGE_2_ than resident KC. In a clinical context, inhibition of PGE_2_ synthesis with non-selective or selective COX inhibitors may enhance inflammation and promote disease progression in diet-induced MASH. Rather than using COX inhibitors as a therapeutic strategy to treat MASLD/MASH.

## Conclusion

Taken together, we could observe a dynamic change in the hepatic macrophage pool during MASLD progression with a decreased ratio of Clec4F^+^Tim4^+^ KC to infiltrating Clec4F^−^Tim4^−^ MoMF and Clec4F^+^Tim4^−^ MoKC. If infiltrating macrophages, which are thought to have a predominantly pro-inflammatory phenotype [43, 72, 73], respond in a similarly sensitive way to PGE_2_ as the “model infiltrating macrophages” PM and BMDM studied here in vitro, the PGE_2_-dependent inhibition of TNF-α formation could attenuate inflammation in the context of MASH. Especially since TNF-α is an early and very potent pro-inflammatory mediator and induces the expression of other pro-inflammatory cytokines such as interleukin-1β and immune cell recruiting chemokines, as well as pro-fibrotic mediators [10, 74]. Given the potential protective role of PGE_2_ during MASH development and in accordance with other studies [62, 63], this may also imply that the therapeutic strategy to treat MASLD with COX inhibitors should be considered with more caution. Instead, a selective modulation of PGE_2_ signaling pathways may hold therapeutic potential.

## Electronic supplementary material

Below is the link to the electronic supplementary material.

Supplementary Material 1

Supplementary Material 2

Supplementary Material 3

Supplementary Material 4

Supplementary Material 5

Supplementary Material 6

Supplementary Material 7

Supplementary Material 8

## Data Availability

No datasets were generated or analysed during the current study.

## Abbreviations

MASLD: metabolic dysfunction-associated steatotic liver disease
MASH: steatohepatitis
KC: Kupffer cells
Cyclooxygenase 1 and 2: COX-1 and COX-2
PGE_2_: prostaglandin E_2_
PM: peritoneal macrophages
BMDM: Bone-marrow-derived macrophages
TNF-α: Tumor necrosis factor-alpha
LPS: lipopolysaccharide
DAMP: danger-associated molecular patterns
mPGES-1: microsomal prostaglandin E_2_ synthase-1
OSM: Oncostatin M (OSM)
MASH-D: MASH-inducing diet
STD: standard diet
Clec4F: C-type lectin domain family 4 member F
Tim4: T cell immunoglobulin and mucin domain containing 4 receptor
MoMF: infiltrating monocyte-derived macrophages
MoKC: monocyte-derived KC
WT: wildtype
KO: knockout
EP1-4: prostaglandin E_2_ receptor 1–4
