# Supplementary material for: Dynamic changes in macrophage populations and resulting alterations in Prostaglandin E2 sensitivity in mice with diet-induced MASH
Source: Cell Commun Signal. 2025 May 16;23:227. doi: 10.1186/s12964-025-02222-y (PMC12083000; doi:10.1186/s12964-025-02222-y)
Supplement: Supplementary file 1 — Supplementary Material 1 [file 12964_2025_2222_MOESM1_ESM.docx]

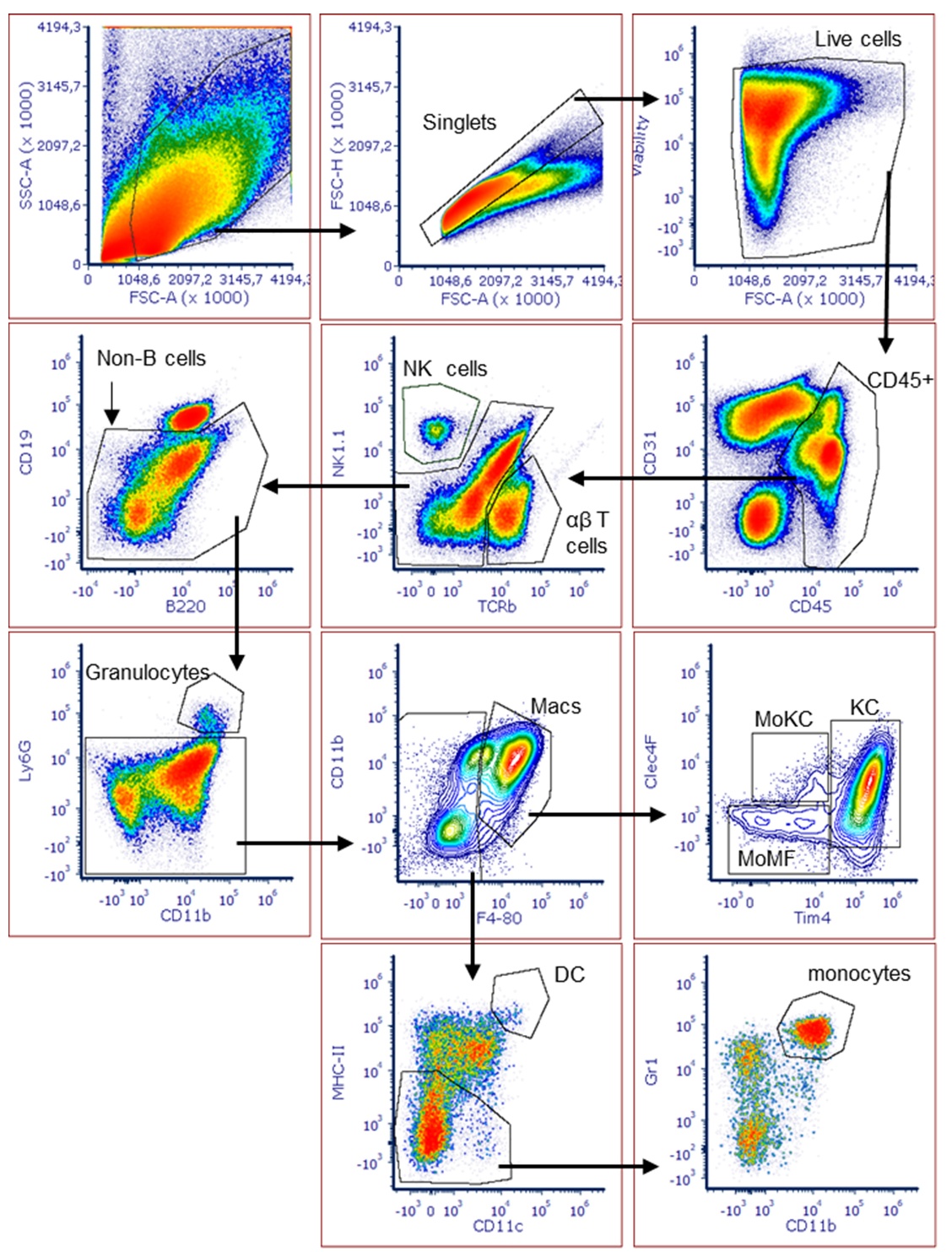


**Figure S1. Gating strategy for liver myeloid cell subsets.** Representative Flow plots showing the gating strategy used for identification of hepatic myeloid cell populations in mice with diet-induced MASH. Abbreviations: Macs: macrophages; KC: Kupffer cells; MoKC: monocyte-derived Kupffer cells; MoMF: monocyte-derived macrophages; DC: Dentritic cells.
