# Supplementary material for: Dynamic changes in macrophage populations and resulting alterations in Prostaglandin E2 sensitivity in mice with diet-induced MASH
Source: Cell Commun Signal. 2025 May 16;23:227. doi: 10.1186/s12964-025-02222-y (PMC12083000; doi:10.1186/s12964-025-02222-y)
Supplement: Supplementary file 2 — Supplementary Material 2 [file 12964_2025_2222_MOESM2_ESM.docx]

**
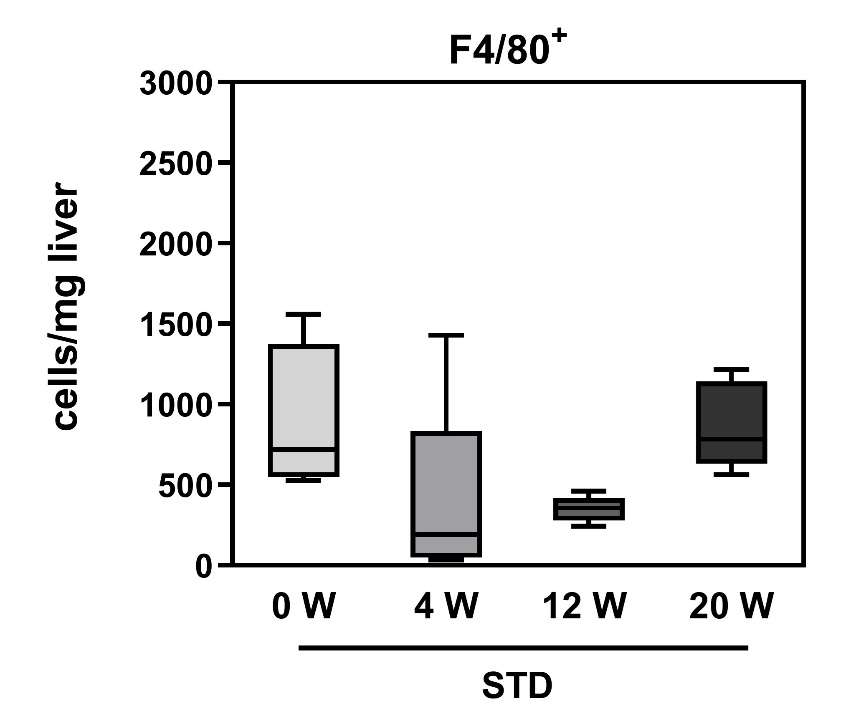
**

**Figure S2. Quantification of F4/80+ cells in livers of STD fed control mice.** Hepatic myeloid cell populations were isolated from mice fed a STD for the indicated feeding periods. After gating on CD45^+^, live and singlet cells, macrophages were identified as F4/80^+^ cells and quantified per milligram liver tissue. Values are median (line), upper- and lower quartile (box) and extremes (whiskers) of n=4 (0 W), n=7 (4 W), n=7 (12 W), n=7 (20 W) mice. Statistics: One-way-ANOVA with Tukey´s *post hoc* test for multiple comparison. **p*<0.05
