# Supplementary material for: Dynamic changes in macrophage populations and resulting alterations in Prostaglandin E2 sensitivity in mice with diet-induced MASH
Source: Cell Commun Signal. 2025 May 16;23:227. doi: 10.1186/s12964-025-02222-y (PMC12083000; doi:10.1186/s12964-025-02222-y)
Supplement: Supplementary file 3 — Supplementary Material 3 [file 12964_2025_2222_MOESM3_ESM.docx]

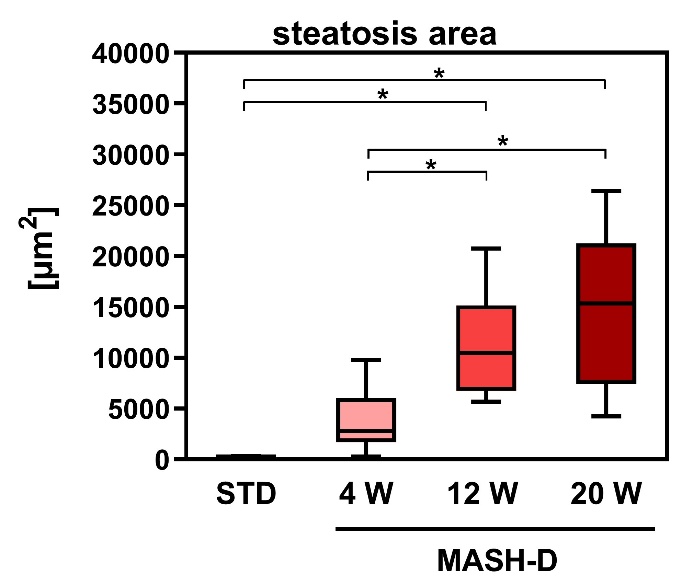


**Figure S3. Diet-dependent changes in hepatic steatosis.** Wildtype mice were fed a standard (STD) or MASH-inducing diet (MASH-D) for 4, 12 and 20 weeks. Lipid droplet area, as a parameter of steatosis, was quantified in parenchymal regions of liver slices stained with Hematoxylin & Eosin. Values are median (line), upper- and lower quartile (box) and extremes (whiskers) of n=7-8 (STD), n=15 (4 W MASH-D), n=12-13 (12 W MASH-D), n=10-12 (20 W MASH-D) mice. Statistics: One-way-ANOVA with Tukey´s *post hoc* test for multiple comparison. **p*<0.05
