# Supplementary material for: Dynamic changes in macrophage populations and resulting alterations in Prostaglandin E2 sensitivity in mice with diet-induced MASH
Source: Cell Commun Signal. 2025 May 16;23:227. doi: 10.1186/s12964-025-02222-y (PMC12083000; doi:10.1186/s12964-025-02222-y)
Supplement: Supplementary file 4 — Supplementary Material 4 [file 12964_2025_2222_MOESM4_ESM.docx]

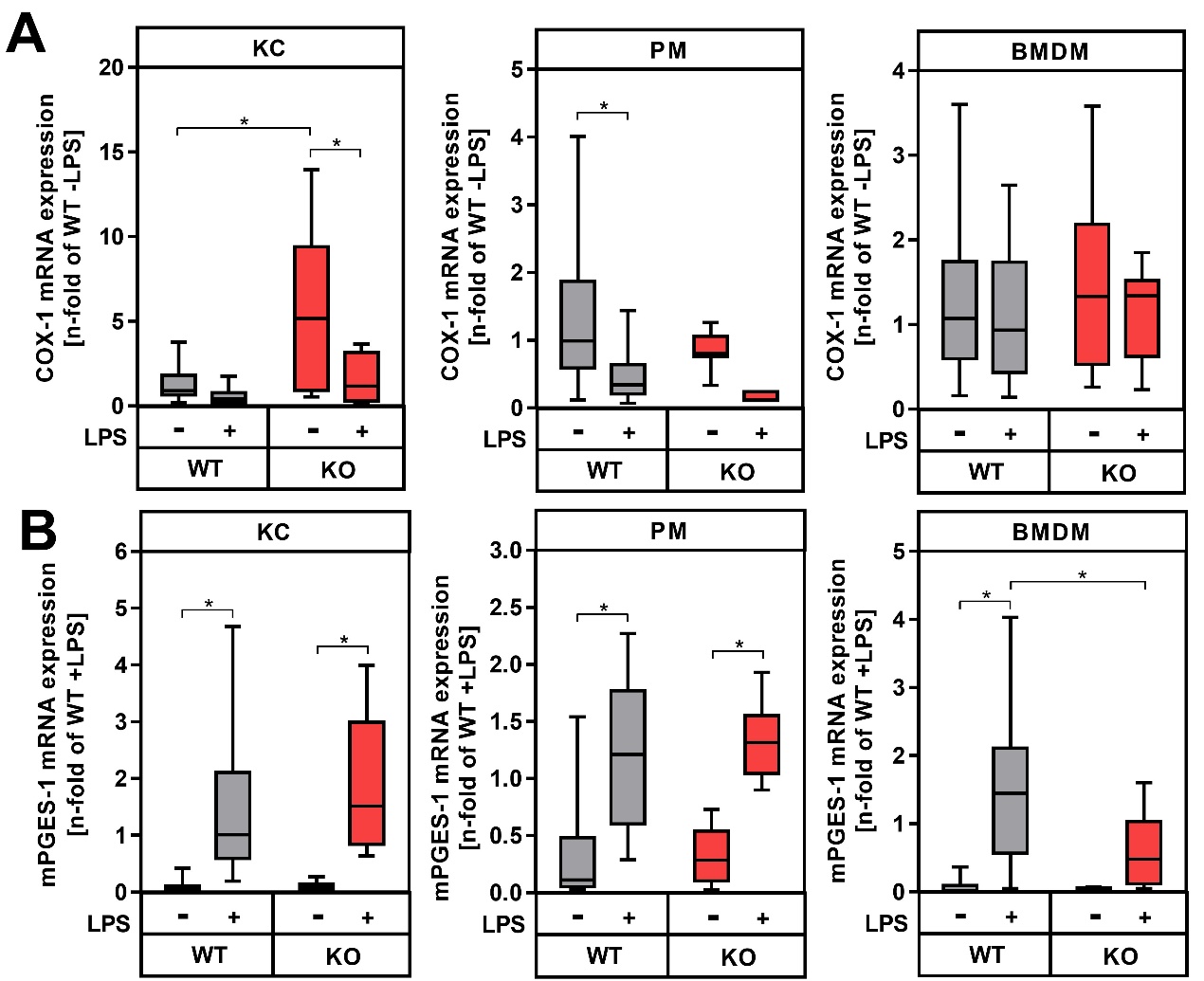


**Figure S4. COX-1 and mPGES-1 mRNA expression in macrophages from wildtype controls (WT) and COX2-deficient mice (KO).** Primary Kupffer cells (KC), peritoneal macrophages (PM) and bone marrow-derived macrophages (BMDM) were stimulated with for 24 h with LPS. Relative mRNA expression of COX-1 (gene name *Ptges1*; A) and mPGES-1 (gene name *Ptges*; B) were determined by RT-qPCR with *Hprt* as the reference gene. Values are median (line), upper- and lower quartile (box) and extremes (whiskers) of n=26-32 (WT) or n=5-10 (KO) independent experiments. Statistics: Two-way-ANOVA with Tukey´s *post hoc* test for multiple comparison. **p*<0.05.
