# Supplementary material for: Dynamic changes in macrophage populations and resulting alterations in Prostaglandin E2 sensitivity in mice with diet-induced MASH
Source: Cell Commun Signal. 2025 May 16;23:227. doi: 10.1186/s12964-025-02222-y (PMC12083000; doi:10.1186/s12964-025-02222-y)
Supplement: Supplementary file 5 — Supplementary Material 5 [file 12964_2025_2222_MOESM5_ESM.docx]

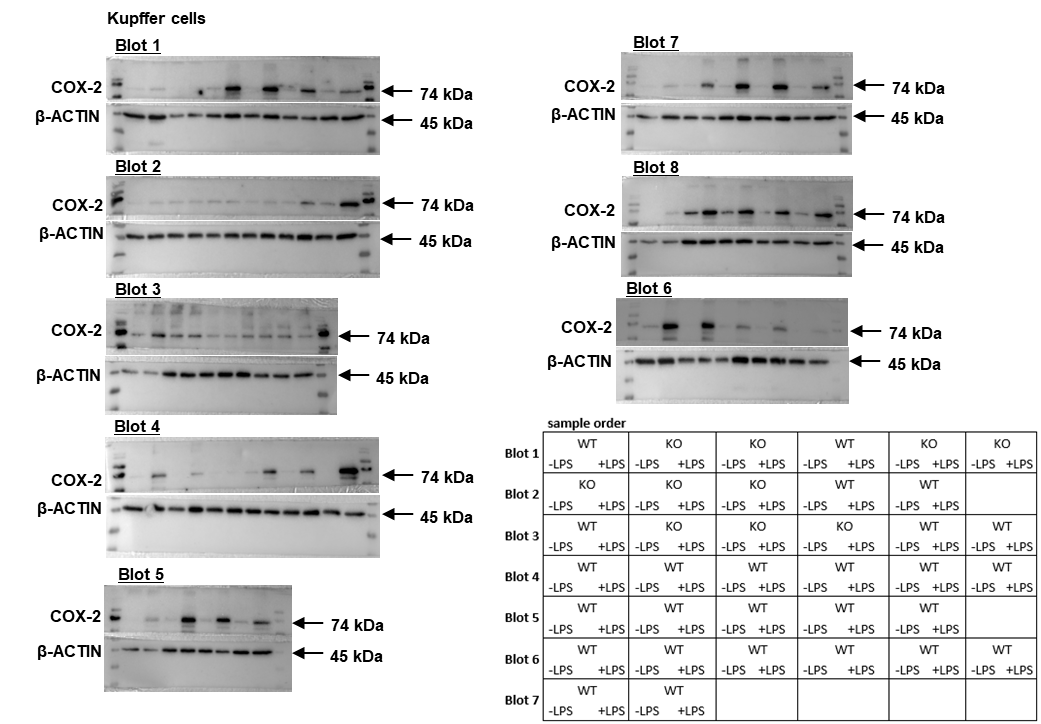


**Figure S5. Detection of COX-2 and β-Actin in Kupffer cells.** Primary Kupffer cells (KC) were stimulated with for 24 h with LPS. Protein lysates were analyzed by immunoblotting for COX-2 protein expression, with β-Actin serving as the loading control.


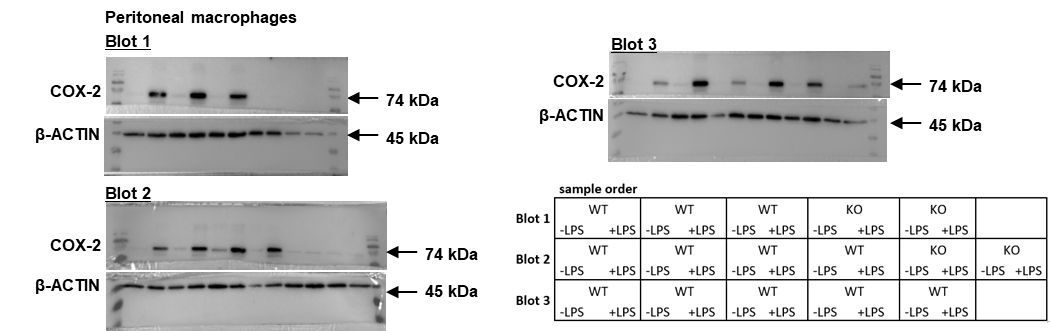


**Figure S6. Detection of COX-2 and β-Actin in peritoneal macrophages.** Primary peritoneal macrophages (PM) were stimulated for 24 h with LPS. Protein lysates were analyzed by immunoblotting for COX-2 protein expression, with β-Actin serving as the loading control.


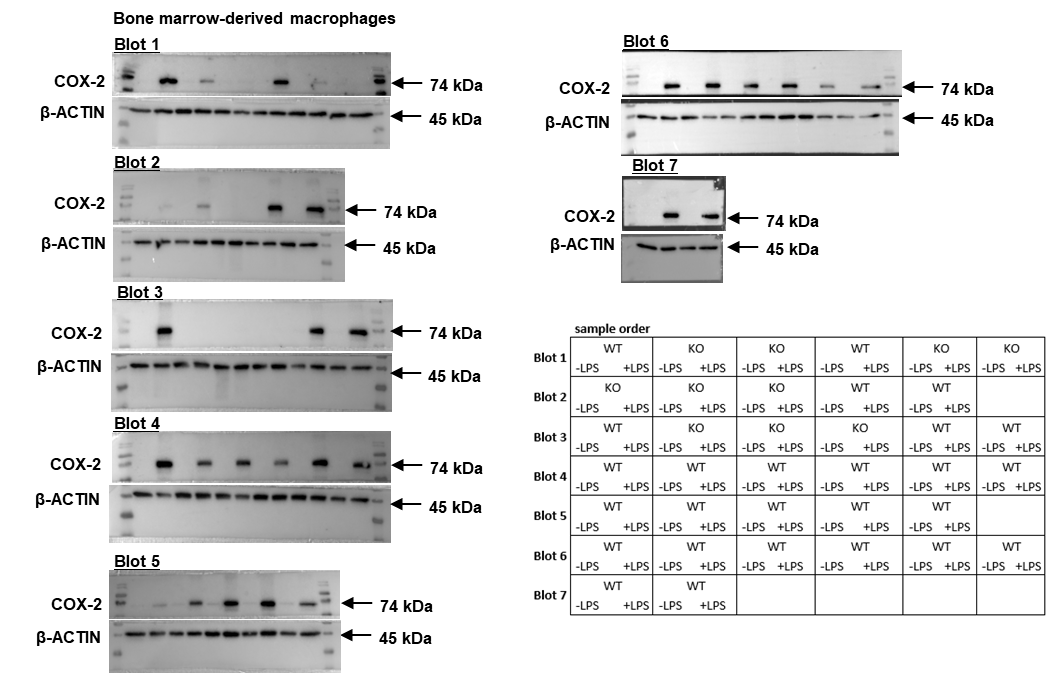


**Figure S7. Detection of COX-2 and β-Actin in bone marrow-derived macrophages.** Primary bone marrow-derived macrophages (BMDM) were stimulated for 24 h with LPS. Protein lysates were analyzed by immunoblotting for COX-2 protein expression, with β-Actin serving as the loading control.
