# Supplementary figures and images for: Dynamic changes in macrophage populations and resulting alterations in Prostaglandin E2 sensitivity in mice with diet-induced MASH
Source: Cell Commun Signal. 2025 May 16;23:227. doi: 10.1186/s12964-025-02222-y (PMC12083000; doi:10.1186/s12964-025-02222-y)

**Supplementary Table T1:** Antibodies used for flow cytometry analysis of hepatic myeloid cells.


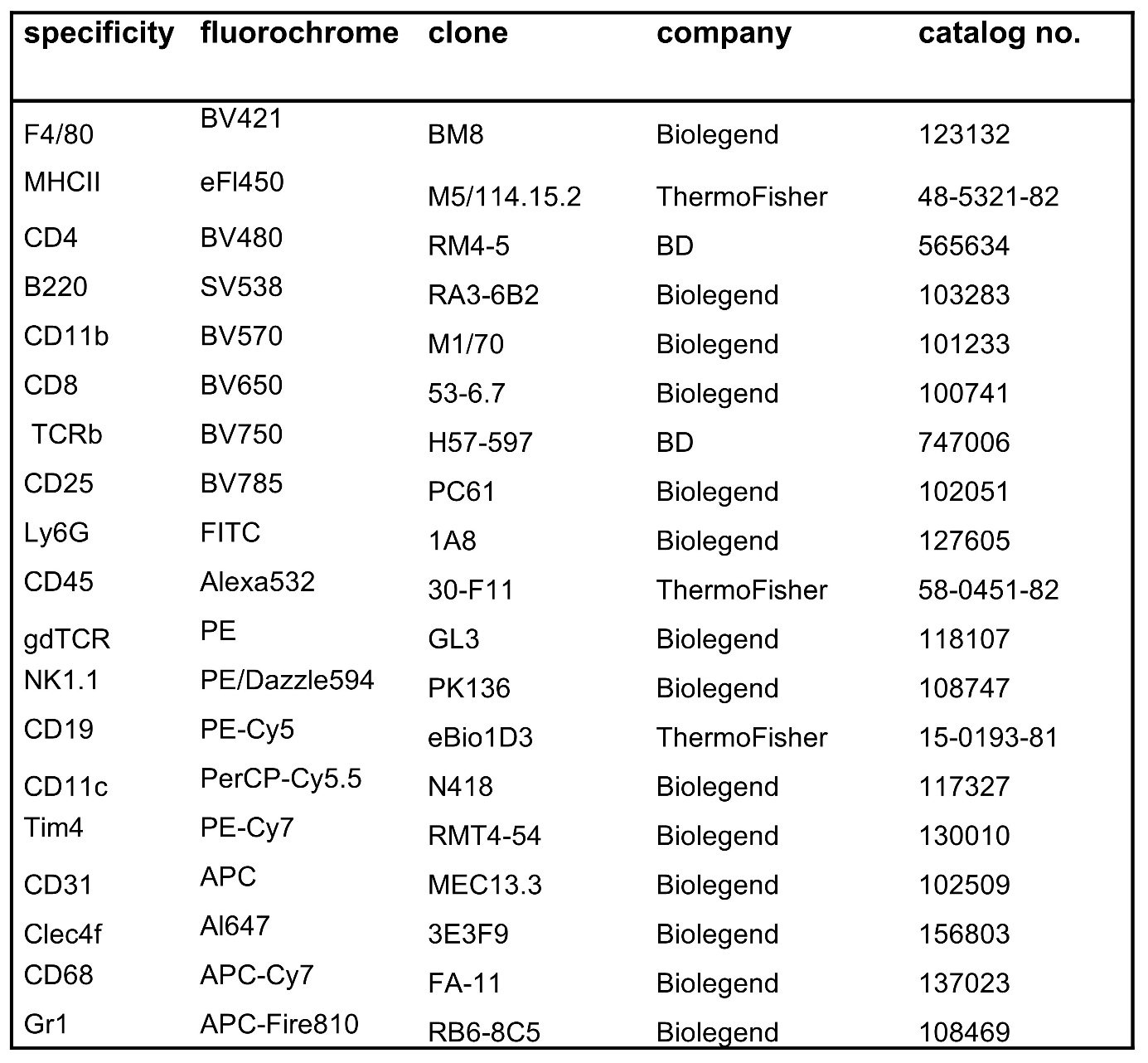

Supplement: Supplementary file 6 — Supplementary Material 6 [file 12964_2025_2222_MOESM6_ESM.docx]
