# Supplementary material for: Dynamic changes in macrophage populations and resulting alterations in Prostaglandin E2 sensitivity in mice with diet-induced MASH
Source: Cell Commun Signal. 2025 May 16;23:227. doi: 10.1186/s12964-025-02222-y (PMC12083000; doi:10.1186/s12964-025-02222-y)

**Supplementary Table T2:** Sequences of the oligonucleotides (Biolegio, Nijmegen, The Netherlands) used for RT-qPCR. The oligonucleotide sequences were derived on the basis of the given accession numbers. They were checked for specificity by BLAST search.


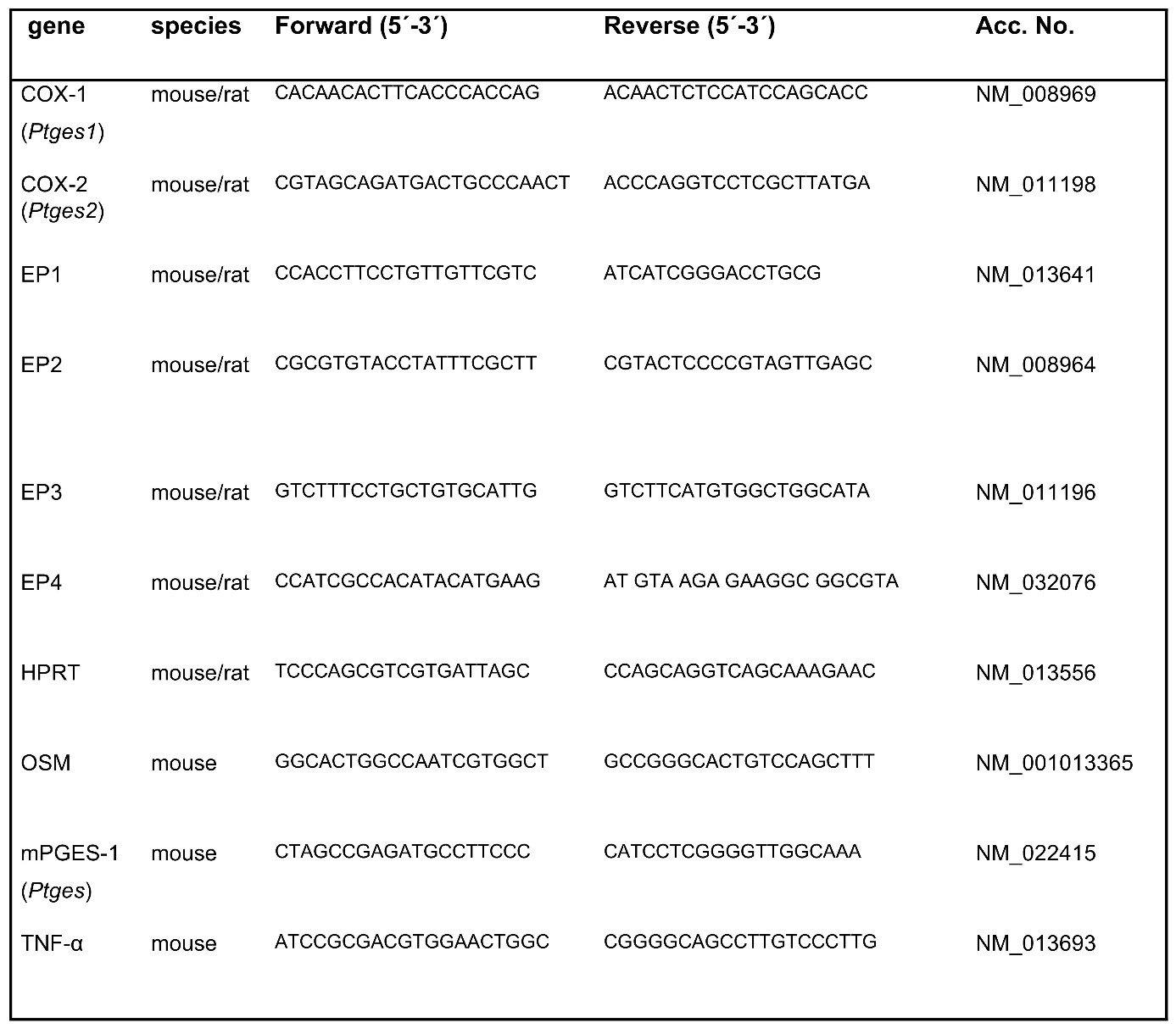

Supplement: Supplementary file 7 — Supplementary Material 7 [file 12964_2025_2222_MOESM7_ESM.docx]
