# Supplementary material for: Dynamic changes in macrophage populations and resulting alterations in Prostaglandin E2 sensitivity in mice with diet-induced MASH
Source: Cell Commun Signal. 2025 May 16;23:227. doi: 10.1186/s12964-025-02222-y (PMC12083000; doi:10.1186/s12964-025-02222-y)

**Supplementary Table T3. Diet-dependent changes in hepatic triglyceride and cholesterol content of mice with diet-induced MASH.** Wildtype mice were fed with a standard (STD) or MASH-inducing diet (MASH-D) for 4, 12 and 20 weeks. Hepatic triglyceride and cholesterol content was quantified in tissue homogenates using a common colorimetric assay. Values are mean ± SEM of n=7 (STD), n=15 (4 W MASH-D), n=12 (12 W MASH-D), n=10-11 (20 W MASH-D) mice. Statistics: One-way-ANOVA with Tukey´s *post hoc* test for multiple comparison. (*) vs. STD and (^#^) vs. 4 W MASH-D with *p*<0.05


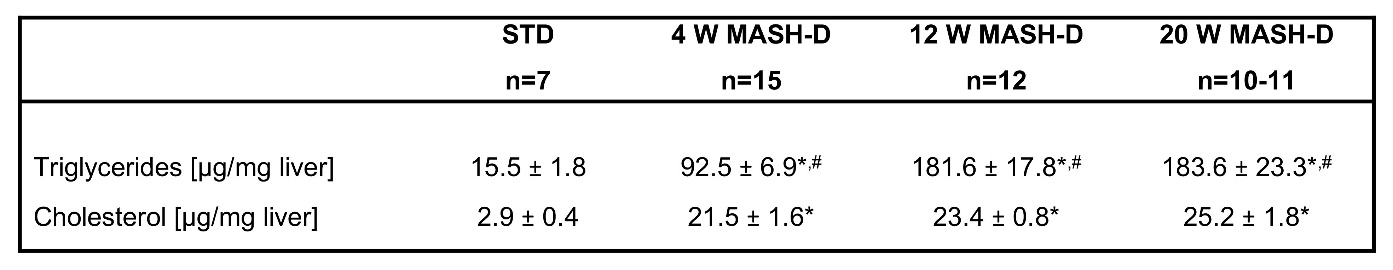

Supplement: Supplementary file 8 — Supplementary Material 8 [file 12964_2025_2222_MOESM8_ESM.docx]
